# Deep Brain Stimulation in Drug Addiction Treatment: Research Progress and Perspective

**DOI:** 10.3389/fpsyt.2022.858638

**Published:** 2022-04-07

**Authors:** Rui Chang, Jionghong Peng, Yunfan Chen, Hailin Liao, Size Zhao, Ju Zou, Sijie Tan

**Affiliations:** Department of Histology and Embryology, School of Basic Medicine, Hengyang Medical College, University of South China, Hengyang, China

**Keywords:** drug addiction, deep brain stimulation, nucleus accumbens, substantia nigra pars reticulata, opioids

## Abstract

Drug addiction is a chronic psychiatric disorder characterized by compulsive drug-seeking and drug-using behavior, and a tremendous socioeconomic burden to society. Current pharmacological and psychosocial methods have shown limited treatment effects for substance abuse. Deep Brain Stimulation (DBS) is a novel treatment for psychiatric disease and has gradually gained popularity in the treatment of addiction. Addiction is characterized by neuroplastic changes in the nucleus accumbens (NAc), a key structure in the brain reward system, and DBS in this region has shown promising treatment effects. In this paper, the research progress on DBS for drug addiction has been reviewed. Specifically, we discuss the mechanism of NAc DBS for addiction treatment and summarize the results of clinical trials on DBS treatment for addiction to psychoactive substances such as nicotine, alcohol, cocaine, opioids and methamphetamine/amphetamine. In addition, the treatment effects of DBS in other brain regions, such as the substantia nigra pars reticulata (SNr) and insula are discussed.

## Introduction

Addiction is a brain disease manifested by compulsive substance use despite harmful consequences. Drug addiction is one of the most serious and global public health problems. According to a recent survey, smoking, alcohol abuse and illegal drug use cause 11.8 million deaths each year, which is more than all cancer deaths (1). At present, several treatment methods for psychoactive substance use disorders have been developed, but the effects are unsatisfactory. In particular, achieving long-term abstinence with medical treatment has been difficult to achieve and pharmacological treatments have been ineffective in reducing drug use or criminal activity (2). It was shown that about 85% of addicts relapse within 1 year after abstinence, and people who did not receive any treatment have a greater risk of mortality (3, 4). Therefore, seeking a safe, effective and convenient treatment for refractory drug addiction is urgently needed.

With the development of neurosurgery, Deep Brain Stimulation (DBS) technology has gradually gained popularity in treating addiction. DBS uses 3D imaging technology to accurately target specific areas of the brain, such as the subthalamic nucleus (STN) and the nucleus accumbens (NAc), using intracranially implanted electrodes connected to pectorally implanted batteries and pulse generators (IPG). During stimulation, DBS electrodes influence neural activity through high-intensity pulses to achieve the desired therapeutic effect. DBS technology was originally used for the treatment of movement disorders starting in the 1980's, and has achieved satisfactory results in the treatment of progressive dyskinesias (5). By 2019, more than 160,000 patients worldwide have undergone DBS for a variety of neurological and non-neurological conditions, and DBS shows positive therapeutic effects (6). For example, the motor symptoms and levodopa-induced involuntary movements of Parkinson's disease patients have been significantly improved by DBS in the subthalamic nucleus (STN) (7).

Compared with traditional brain neurosurgery, DBS has the characteristics of reversibility and controllability, and is regarded as a safe alternative treatment compared to pharmacological treatment for neurological and psychiatric conditions. So far, the Food and Drug Administration (FDA) has approved DBS technology for Parkinson's disease (PD), obsessive-compulsive disorder (OCD), essential tremor (ET) and primary dystonia (8). In addition, tremendous efforts have been devoted to explore the therapeutic effects of DBS for neuropsychiatric diseases. Major progress has been made in the development of DBS as a treatment for refractory substance use disorders. In a study of alcohol addiction treatment with DBS, 2 out of 5 patients remained abstinent for many years, and 3 patients showed a significant reduction in alcohol consumption during the 8 years follow-up (9). Preclinical studies also show reduced relapse to cocaine and heroin seeking after NAc DBS (10–12). Therefore, DBS may become one of the most promising treatments for substance use disorders. However, the current research on the effects of DBS on drug addiction has only been conducted in limited clinical studies (13), and more trials are needed to further verify its effectiveness (14). In this review, we discuss the mechanism of DBS for addiction treatment and summarize the limited research on the application of DBS for the treatment of addiction to psychoactive substances such as nicotine, alcohol, cocaine, opioids and methamphetamine/amphetamine. The goal of the current review is to summarize the existing literature on the effects of DBS treatment for drug addiction to help advance our understanding of the neural mechanisms responsible for addiction and provide inspiration for continued clinical trials.

## Neurobiology of Drug Addiction

The etiology and pathogenesis of drug addiction are related to altered functioning of multiple brain systems. These include altered glutamate, opioid, cannabinoid, gamma-aminobutyric acid, norepinephrine and serotonergic systems. In addition, the mesolimbic dopaminergic reward system plays a central role in the pathogenesis of addiction and hypofunction of this system is a key characteristic of drug addiction. It is widely accepted that the initial reinforcing effects of drugs of abuse are mediated by large and rapid increases in the level of dopamine (DA) in the NAc. However, prolonged use of these drugs leads to hypofunction of the dopamine system (15). It has been hypothesized that DBS may alleviate addiction symptoms through normalizing dopamine levels and restore the functioning of the reward system (2).

Common addictive substances include nicotine, alcohol, cocaine, methamphetamine, marijuana, opioids, tranquilizers and sedatives (16), and the neurobiological mechanisms that produce addiction to these substances have been extensively investigated. So far, all known drugs of abuse increase dopamine in the NAc either by (1) the activation of dopamine neurons, or by (2) enhancing dopamine neurotransmission through facilitated release from terminals or blocking reuptake. For instance, studies have reported that the reinforcing properties of alcohol are closely related to the release and transmission of dopamine in the NAc. In particular, it was found that alcohol acutely increases the concentration of extracellular dopamine by 25% to 50% (17). Opioids are another commonly abused class of drugs that includes natural products obtained from opium poppy, and synthetic drugs with similar pharmacological properties (18, 19). Studies have found that the rewarding properties of opioids are mediated by enhancing the activity of dopamine neurons in the ventral tegmental area (VTA) and increasing the release of dopamine in the NAc (20). Physical dependence is common in opioid addicts and characterized by insomnia, cramps, diarrhea, nausea, vomiting and body pain, as well as irritability and anxiety when opioid use is suddenly stopped (21). The negative reinforcement effect of avoiding these withdrawal symptoms is part of the reason for continued use of opioids (22).

Addiction caused by these psychoactive substances is considered to be a relapse disorder with repeated stages. It is a cycle composed of three stages of “drug use,” “withdrawal” and “craving and relapse”(15), and animal and human imaging studies have revealed the neural circuits that mediate the three phases of the addiction cycle. The VTA and striatum play a key role in the drug use phase, and the extended amygdala plays a key role in the withdrawal phase. The orbital frontal cortex-dorsal striatum, pre-frontal cortex, basolateral amygdala, hippocampus and insula play a key role in the craving phase (22). Therefore, DBS may affect the release of neurotransmitters and neuronal firing by affecting the key nuclei in these circuits, thereby reducing craving and relapse.

## The Mechanism of DBS Treatment in Psychiatric Disease

At present, the specific mechanism that underlie the therapeutic effects of DBS for the treatment of psychiatric disease has not been fully understood, but great progress has been made in the treatment for obsessive-compulsive disorder (23, 24), major depressive disorder (25), Tourette's syndrome (26) and other neurological disorders such as Parkinson's disease (27). DBS is thought to produce therapeutic effects through the following mechanisms.

### Inhibition of Local Neurons

DBS was initially thought to inhibit local neurons because the therapeutic effects of DBS in the subthalamic nucleus were similar to the effects of damage to the subthalamic nucleus (28, 29). However, the discharge frequency of globus pallidus neurons was found to be significantly increased when DBS was applied to the subthalamic nucleus of rhesus monkeys, suggesting that DBS is capable of activating regions connected to the target site (30). Dostrovsky and Lozano put forward a view based on synaptic inhibition that DBS stimulation leads to the release of GABA in basal ganglia structures characterized by dense GABAergic input, thereby inhibiting downstream neurons (31). Research results of Feuerstein et al. (32) analyzed the physiological commonality of the target site stimulated under different neuropsychological states, and proposed that the selective release of GABA can fully explain the effects and side effects of DBS. Urbano et al. (33) proposed a synaptic depletion hypothesis which indicated that high-frequency stimulation may cause transmitter depletion, thereby preventing the efferent output of stimulated neurons. Therefore, DBS may inhibit neurons in the target region and promote activity in connected regions.

### Changing Neuroplasticity

Neuroplasticity is the basis of learning and long-term memory. DBS of the anterior thalamic nucleus and ventromedial pre-frontal cortex of rats can promote the proliferation of granule cells in hippocampal dentate gyrus and the increase of hippocampal synaptic density, respectively (34). Treating cocaine addicted mice with DBS and dopamine receptor blockers can normalize the synaptic remodeling produced by cocaine exposure (35).

### Interruption of Pathological Neural Oscillations

Abnormal neural oscillations will affect the communication function of the brain. Rhythmic DBS interrupts the pathological neural oscillations in the target nucleus and connected circuit, and normalizes dysfunctional information flow to improve brain function (36). The impact of DBS has also been explained at the neural network rather than the cellular level (37). Interestingly, high-frequency DBS not only produces region-specific alterations in local field potential (38), but also interrupts dysfunctional network states through both orthodromic and antidromic activation of axons near the electrode (39). High-frequency DBS of the NAc suppresses neuronal activity and selectively modulates afferent drive in the orbitofrontal cortex in rats (40). Hammond et al. (41) proposed that DBS can correct the spontaneous pathological mode of a neuron network and inferred that extracellular stimulation activates subpopulations of afferent and efferent axons.

Addiction to psychoactive substances, also known as psychoactive substance use disorder, is characterized by repeated desire to continue taking the substance regardless of harmful consequences (42). Although several treatments including psychotherapy, behavior modification and drug therapy have been applied for addiction (43), the recurrence rate is as high as 50–70% (14). Therefore, there is an urgent need for a new treatment method to improve the symptoms of substance use disorder and reduce its relapse rate. In a previous review of the literature, Luigjes and colleagues summarized all potential targets of DBS for substance use disorder, and found that seven animals studies had been conducted in six brain regions: NAc, STN, dorsal striatum, lateral habenular nucleus, medial pre-frontal cortex (mPFC) and hypothalamus, and 11 human studies targeting two brain regions (NAc and STN). The authors concluded that the NAc is the most promising DBS target for the treatment of patients with refractory addiction (44). For example, in a study of six severe alcohol dependence patients treated with bilateral high-frequency NAc DBS, the patients showed significant reduction in alcohol craving and alcohol consumption, and the patients stopped drinking and alcohol craving remained reduced 1 year after the initial treatment (45). In addition, the NAc has also emerged as a common target for the comorbidity of depression and addiction, highlighting a common role of NAc in psychiatric diseases (46).

Nevertheless, the mechanism of NAc DBS treatment for addiction is still unclear. Some animal studies indicate that the reduction of addictive behavior depends on the normalization of activity in the NAc shell, a part of NAc responsible for the perception of the rewarding effects of drugs (47, 48). Additionally, It has been shown that the effects of NAc shell DBS depend on altering upstream activity in the pre-frontal cortex (38, 40, 47, 49). Creed and colleagues (35) found that while DBS reduced cocaine sensitization with typical parameters (130 Hz), it was only effective at reversing cocaine induced neuroplasticity in the presence of a dopamine D1 receptor antagonist (and with low frequency 13 Hz stimulation), suggesting that the mechanism of DBS treatment for addiction may be mediated through changes in neuroplasticity. Interestingly, a recent study found that DBS of the NAc shell induced a slight increase in cocaine self-administration and decreased irritability-like behavior during cocaine withdrawal, suggesting DBS might not decrease cocaine intake in active, long-term cocaine users, but might be useful for the treatment of the negative emotional state that emerges during cocaine abstinence (50). Moreover, NAc DBS has also been shown to reduce both sucrose and cocaine seeking, suggesting it might produce non-specific effects on motivation (51).

## The Application of DBS in Addiction to Psychoactive Substances

Since 2003, several clinical trials of DBS treatment in addiction to psychoactive substances such as tobacco, alcohol, cocaine, opioids and methamphetamine/amphetamine have been completed. The stimulation parameters, outcome, duration of follow-up and reported side effects have been summarized in Table 1. All of the reported NAc DBS trials showed positive treatment effects on addiction. So far, all clinical trials have been small scale, and much of the clinical data comes from case reports.

**Table 1:** Human DBS trials for addiction.

**References**	**Country**	**Drug**	**Numbers of participants**	**Target**	**Stimulation parameters**	**Outcome**	**Duration of follow-up**	**Side effects**
Muller et al. (9)	Germany	Alcohol	5	NAc	130 Hz, 90μs, 3.5–4.5 V	2 patients remained abstinent for many years and 3 patients showed a marked reduction of alcohol consumption	up to 8 years	No severe or long-standing side effects occurred.
Kuhn et al. (45)	Germany	Alcohol	1	NAc	130 Hz,120 us, 5.5V	DBS of the NAc led to a significant reduction of drug consumption and modulated associated deficits in cognitive control	1 year	Not reported
Voges et al. (52)	Germany	Alcohol	5	NAc	130 Hz,4.5 V,90 μs	All patients experienced significant and ongoing improvement of craving. Two patients remained completely abstinent for more than 4 years.	38 months	NAc stimulation was tolerated without permanent side effects
Kuhn et al. (53)	Germany	Tobacco	10	NAc	130–145 Hz, 90 us, 180 us, 3–6V	Three male patients were able to quit smoking after DBS	1-year, 2-year, and 30-month	Tolerated without permanent side effects.
Mantione et al. (54)	Canada	Tobacco	14	NAc	180 Hz, 90 us, 3.5 V	substantial clinical improvement after DBS treatment	8 months	Five patients reported forgetfulness.
Goncalves et al. (55)	Italy	Cocaine	1	NAc	150 Hz, 150 μs, 3–4 V/2.5–3 V	The patient's subjective impression was of a marked positive effect of continuous DBS in increasing his control over cocaine craving and intake.	2.5 years	No severe or permanent adverse events occurred.
Zhang et al. (56)	China	Methamphetamine	1	NAc	130 Hz, 210 us, 90 us, 2.5–3v	Successfully treated using DBS of the nucleus accumbens and ventral capsule.	1 year	No significant side effects or complications
Kuhn et al. (57)	Germany	Opioids	2	NAc	140 Hz, 120 us, 5 V (*n* = 1) 130 Hz, 90 us, 4.5 V (*n* = 1)	Amelioration of the patients' depressive and anxious symptoms and increase of the subjectively perceived quality of life	2 years	Lacking alternative behavioral patterns and compensatory social skills.
Valencia-Alfonso et al. (58)	Netherlands	Opioids	1	NAc	180 Hz, 90 us, 4.5 V	Patient is currently clean for more than 6 months	6 months	a 14-day relapse
Zhou et al. (59)	China	Opioids	1	NAc	145 Hz, 90 us, 2.5 V	There was no relapse during the 6-year follow-up	6 years	No severe or long-standing side effects occurred.
Ge et al. (60)	China	Methamphetamine/ Amphetamine	2	NAc	150 Hz, 210 us, 2.5 V (*n* = 1) 165 Hz, 240 us, 3.3 V	One patient (A) remained abstinent and presented with positive emotional experiences, while the other (B) had no significant psychobehavioral changes during stimulation at low to moderate voltages and subsequently relapsed.	2 years	One patient relapsed

*DBS, deep brain stimulation; NAc, nucleus accumbens*.

### Tobacco

The effect of DBS on smoking cessation was unexpectedly found in a study of DBS treatment for other neuropsychiatric disorders. Among patients with anxiety disorder, obsessive-compulsive disorder or Tourette syndrome with a long-term smoking history, three out of every 10 people were able to quit smoking after DBS, and the smoking addiction severity and cigarette consumption of the remaining seven participants also decreased significantly during 1-year, 2-year, and 30-month follow-up. Kuhn et al. (53) found that the success rate of smoking cessation (20, 30 and 30%) was also higher than the success rate of smoking cessation without DBS treatment (1.3, 1.9 and 8.7%). And in another study, it was also found that NAc DBS significantly reduced the craving and consumption of cigarettes in a single subject who initially received DBS treatment for refractory OCD (54).

### Alcohol

To date, three studies investigated the effect of NAc DBS on alcohol use and found that alcohol intake or craving was reduced in all the studies (9, 45, 52). In the study by Voges et al. (52), five male patients aged 36–65 years old were treated with the above-mentioned bilateral NAc DBS to treat severe alcohol addiction as defined by ICD-10 and DSM-IV standards. It was found that two out of the five patients were able to achieve long-term (>4 years) abstinence, and that the alcohol cravings of the other three patients were also significantly reduced. Accordingly, in two other studies (9, 45), six patients received bilateral high-frequency NAc DBS treatment for severe alcohol dependence, and it was found that all six patients showed significantly reduced alcohol craving and alcohol consumption. Moreover, no permanent side effects were observed with NAc DBS (52).

### Cocaine

In 2016, Gonzales-Ferreira treated a cocaine addicted patient using DBS electrodes placed in the medial NAc adjacent to the bed nucleus of the stria terminalis (BNST) (55). Six months after the operation, cocaine use and craving for cocaine were significantly reduced in this patient. And the abstinence was achieved at the 2-year follow up (55). Moreover, in this patient no differences were observed between blind DBS ON and OFF conditions, which may indicate a placebo effect or changes in synaptic plasticity produced by DBS (55).

### Opioids

Recently, three studies investigated the effects of NAc DBS on opioid consumption or cravings (57–59) and reported significant reductions in opioid use and craving after treatment. Kuhn et al. (57) found that NAc DBS reduced the cravings of two drug-resistant patients and induced heroin withdrawal. In another patient with heroin dependence, bilateral NAc DBS significantly reduced his intake and craving for heroin within 6 months after the operation (58). In addition, in a study on a 28-year-old patient with polysubstance use disorder (bucinnazine, morphine, and hypnotics) for 13 years, the craving for the three drugs decreased markedly and he remained abstinent throughout the follow-up period of approximately 1-year after bilateral NAc DBS combined with bilateral anterior capsulotomy (61).

### Methamphetamine/Amphetamine

Zhang et al. (56) found that patients with methamphetamine addiction after NAc/ventral capsule (VC) DBS treatment stopped taking methamphetamine 1 year after the operation, and their social function also improved. Ge et al. (60) reported the results of NAc DBS treatment on two methamphetamine (MA) addicted patients. During the ~2-year follow-up period, one patient remained abstinent, whereas the other had no significant psychobehavioral changes during stimulation at low-to-moderate voltages and subsequently relapsed, and the reason was attributed to misplacement of the DBS electrode.

## Conclusion and Perspective

Drug addiction is a chronic mental disorder characterized by compulsive drug-seeking and use behavior. Substance use disorders are a major socioeconomic burden to society. In recent decades, DBS has emerged as an effective way to treat addiction to psychoactive substances, providing a novel option for patients with high relapse risks after traditional treatments have failed.

Although the NAc is the most widely used site for the stereotactic implantation of DBS electrodes for the treatment of addiction (61, 62), therapeutic effects have also been reported in other brain regions. Preclinical data demonstrated that DBS of the infralimbic cortex, the brain region sending major glutamatergic projections to the NAc attenuated cocaine-primed reinstatement of drug seeking in rats (63). In another preclinical study, Fakhrieh-Asl and colleagues (64) reported that DBS of the OFC prevented morphine conditioned place preference (CPP), facilitated extinction of morphine CPP, and blocked drug priming-induced reinstatement of morphine seeking in rats. In addition, Ibrahim and colleagues (65) proposed the insula, a region of the cerebral cortex involved in critical aspects underlying substance use disorders, such as decision making, anxiety, cognition, mood, as a promising DBS target of addiction. Moreover, although the SNr is not commonly thought of as a component of the neural circuits that regulate drug seeking, several studies suggesting that SNr may serve as a potential therapeutic target for drug addiction. The potential role of SNr in addiction has also been documented in recent studies. It was found that activation of SNr-GABA neurons prevented cocaine CPP (66). However, Galaj and collegues found that optogenetic activation of SNr-GABA neurons increased heroin self-administration (67). Interestingly, DBS of the substantia nigra pars reticulata (SNr) promoted extinction and prevented reinstatement of methamphetamine-induced CPP, suggesting SNr might be a potential therapeutic target for the treatment of methamphetamine addiction (68). It should be mentioned that the frequency might be important for SNr DBS because high frequency DBS produced therapeutic effects on the extinction and reinstatement of methamphetamine seeking, while slow frequency stimulation impaired extinction (68). Future studies, in particular clinical trials, are needed to explore the effectiveness of these brain regions as a target for addiction treatment.

It should be mentioned that DBS is an invasive surgical intervention with potential risks, including hemorrhage and infection (69, 70). Alternative non-invasive neuromodulation methods, such as transcranial direct current stimulation (tDCS) and repetitive transcranial magnetic stimulation (rTMS), have also shown promise as treatments for drug addiction (71–73). In particular, rTMS has been shown to reduce heroin and methamphetamine craving (74, 75). Interestingly, repeated NAc ultrasound stimulation also suppressed morphine CPP, suggesting NAc ultrasound stimulation might also be applied as a potential non-invasive therapeutic option in treating addiction (76). However, the effectiveness of these non-invasive methods in drug addiction treatment needs to be verified with larger clinic trials.

In conclusion, the application of DBS treatment for drug addiction is challenging but promising. However, it should be mentioned that most results are obtained from case reports. Further double-blinded studies and clinical trials, potential synergy with other non-invasive interventions, and larger patient populations are needed for validating DBS as a standard treatment option for refractory substance use disorders.

## Author Contributions

RC, JZ, and ST wrote the paper. JP, HL, YC, and SZ collected the literature. All authors contributed to the article and approved the submitted version.

## Funding

This work was supported by grants from the National Science Foundation of China (NSFC 81301144) and Research Learning and Innovative Experiment Project for University Students of University of South China (210XCX486, 210XCX510, and 200XCX322).

## Conflict of Interest

The authors declare that the research was conducted in the absence of any commercial or financial relationships that could be construed as a potential conflict of interest.

## Publisher's Note

All claims expressed in this article are solely those of the authors and do not necessarily represent those of their affiliated organizations, or those of the publisher, the editors and the reviewers. Any product that may be evaluated in this article, or claim that may be made by its manufacturer, is not guaranteed or endorsed by the publisher.
